# Integrated treatment of depression and moderate to severe alcohol use disorder in women shows promise in routine alcohol use disorder care – a pilot study

**DOI:** 10.3389/fpsyt.2025.1473988

**Published:** 2025-02-06

**Authors:** Anna Persson, Daniel Wallhed Finn, Alice Broberg, Amanda Westerberg, Åsa Magnusson, Olof Molander

**Affiliations:** ^1^ Department of Clinical Neuroscience, Center for Psychiatry Research, Karolinska Institutet, Stockholm, Sweden; ^2^ The Stockholm Center for Dependency Disorders, Region Stockholm, Stockholm, Sweden; ^3^ WeMind Psychiatry, Stockholm, Sweden

**Keywords:** major depressive disorder, alcohol use disorder, investigational therapies, community mental health services, substance abuse treatment centers

## Abstract

**Introduction:**

Major depression and alcohol use disorder affect millions of individuals worldwide and cause significant disability. They often occur together, and their co-occurrence is associated with more negative outcomes than each disorder on its own. Yet, there is a lack of knowledge on how to best treat co-occurring depression and alcohol use disorder. A pilot study was conducted to investigate the feasibility, credibility, patient satisfaction, preliminary effect, and potential negative effects of an integrated treatment for depression and alcohol use disorder, which has shown promising results in an earlier pilot trial.

**Methods:**

The study was conducted at an outpatient unit in Stockholm, Sweden. Women (n=7) with current depression and alcohol use disorder were offered integrated group treatment that included evidence-based treatment for depression and alcohol use disorder. Criteria for feasibility were based on an earlier study, treatment credibility was measured using the Credibility/Expectancy Questionnaire and patient satisfaction with the Client Satisfaction Questionnaire.

**Results:**

Feasibility, credibility, and patient satisfaction were high. Depression symptom severity and alcohol consumption decreased from baseline to follow up. Negative effects were reported in terms of increased adverse emotional experiences.

**Discussion:**

The investigated integrated treatment for co-occurring depression and alcohol use disorder continues to show promise. Randomized clinical trials are needed to evaluate its effectiveness.

## 1 Introduction

A major depressive episode is characterized by depressed mood and loss of interest or pleasure (1). The lifetime prevalence of a major depressive episode is estimated to be 14.6 percent in high income countries (2), and even higher in Sweden: 23 percent among Swedish men and 31 percent among Swedish women (3). In 2016, major depressive disorder (MDD (1); occurred among more than 167 million individuals globally, and caused in excess of 34 million years lived with disability (YLD’s) and disability-adjusted life-years (DALY’s) (4, 5). It was the fifth leading cause of YLD’s globally (5).

Alcohol use disorder (AUD) is a problematic pattern of alcohol use which leads to clinically significant distress or impairment (1). A World Mental Health Survey found an estimated lifetime prevalence of AUD of 10.8 percent (6), while 10.34 percent of the Swedish population are estimated to have AUD (7). More than 100 million individuals were estimated to suffer from AUD globally in 2016, and AUD caused more than 10 million YLD’s (5), and over 16 million DALY’s (4).

MDD and AUD often co-occur and their co-occurrence has remained stable over time (8–10). Co-occurring MDD and AUD are associated with greater disability, increased suicidal ideation, poorer prognosis and greater health care consumption than each disorder on its own (8, 11–13). US American data shows that 32.8 percent of those who sought treatment for AUD in the past 12 months also suffered from depression (14). Yet, there is a dearth of research on treatment of co-occurring depression and alcohol misuse (15, 16). Cognitive behavioral therapy (CBT) and motivational interviewing (MI) in combination have been shown to have a small but significant effect compared to treatment as usual, but more research on how to best treat co-occurring depression and AUD is needed (15, 16).

An integrated treatment based on evidence-based treatment for co-occurring MDD and substance use disorders (SUD, including AUD), has been developed and tried in Swedish outpatient healthcare with promising results (17). The treatment, a type of CBT, includes behavioral activation for depression and relapse prevention for SUD (17). We adapted this treatment to treat MDD and AUD specifically.

The aim of the current study was to investigate if group treatment for co-occurring MDD and moderate to severe AUD in women, could be conducted at an outpatient unit within the Swedish public healthcare system. We predicted that the treatment would be feasible, perceived as credible, that the patients would be satisfied with the treatment, and that depression symptom severity and alcohol consumption would be reduced post compared to pre-treatment. We also expected that the patients would report negative effects of the treatment, as research suggests that happiness and self-esteem initially decrease in the first months after resolving alcohol and other drug problems before stabilizing and subsequently increasing over the years (18).

## 2 Materials and methods

A pilot study, with a small number of participants, was conducted to evaluate treatment feasibility, credibility, patient satisfaction, preliminary effects and potential negative effects.

### 2.1 Measures

Good feasibility was defined as:

- Providing the treatment in routine outpatient AUD care.
- Being able to offer a majority (> 85%) of the patients with MDD and moderate to severe AUD the treatment.
- The proportion of sessions completed by participants should be similar to, or higher, than previously reported (17).
- The proportion of participants who completed treatment (all treatment sessions) should be similar, or higher than previously reported (17).
- The proportion of participants who dropped out from treatment should be similar, or smaller than previously reported (17).

Treatment credibility was measured using the Credibility/Expectancy Questionnaire (CEQ) (19), at the first treatment session. Patient satisfaction was measured with the Client Satisfaction Questionnaire (CSQ) (20), at baseline and follow ups.

To investigate preliminary effects, two primary measures were used. The Patient Health Questionnaire (PHQ-9) was used to measure depression symptom severity (21) at baseline, each session and follow ups. The Time Line Follow Back (TLFB) (22) was used to measure alcohol use at baseline, each session and at follow ups. The TLFB gives an estimate of an individual’s alcohol use during a set period of time and has been shown to work well for periods of up to a year (23). From the TLFB two alcohol use measures were derived, grams of alcohol consumed per week as a primary measure, as well as heavy drinking days (HDD), i.e., percentage of days consuming >40 grams of alcohol. In addition to HDD, the blood biomarker phosphatidylethanol (PEth) (24) was used at baseline and follow ups as a secondary measure of alcohol use.

The Negative Effects Questionnaire (NEQ) (25) was used to investigate potential negative events and effects of the treatment at follow ups.

The MINI International Neuropsychiatric Interview, Swedish Translation Version 7.0.0 (MINI-7) (26), was used to assess mental disorders at baseline and follow ups.

### 2.2 Participants and study site

The study was conducted at a publicly funded outpatient unit for women with moderate to severe AUD in Stockholm, Sweden. Patients at the unit are routinely offered psychiatric assessment, evidence-based treatment of AUD and co-occurring psychiatric disorders as well as regular collection of blood and urine samples to follow biomarkers of substance use. Patients with SUD other than AUD are offered referrals to units specializing in SUD. All current patients who had been diagnosed with DSM-5 (1) MDD and DSM-5 (1) moderate to severe AUD, were offered the chance to participate in the study; provided that the following inclusion criteria were fulfilled and that none of the exclusion criteria were. Inclusion criteria were: current DSM-5 (1) MDD and current DSM-5 (1) moderate to severe AUD. Exclusion criteria were: high risk of suicide or homicide; severe self-harm; ongoing intimate partner violence; no wish to reduce alcohol consumption or abstain from alcohol; current psychosis; medical emergencies; cases where the patient’s psychiatrist or psychologist deemed it would not be in the patient’s best interests to participate in the study, e.g. undergoing treatment for cancer with increased risk for infection. Potential participants were given verbal and written information about the study by their psychiatrist. Written informed consent was then obtained from all participants. No potential participants were excluded due to exclusion criteria. In total twelve patients were asked to participate, of whom three declined. Of the remaining nine, eight patients attended the first treatment session and were included in the study. One patient dropped out after session 1 and was excluded from the analyses. All remaining participants completed baseline and post measures. Six participants completed self-report measures at three-month follow-up, and three participants the six-month follow-up. In addition, PEth was taken as part of the clinical routine at the outpatient unit. See **Table 1** for baseline participant characteristics. The study was conducted in accordance with the Declaration of Helsinki and approved by the Stockholm Ethical Review Board. Participation in the study did not affect the patients’ care. Recruitment took place in the autumn of 2017 and the last follow-up measure was administrated in the spring of 2018.

**Table 1:** Participant characteristics at baseline.

	Total (N=7)
Age	
Age, years, M (SD)	52.9 (8.6)
MDD and alcohol use history	
Age at depression debut, M (SD)	28.3 (14.6)
Age at debut of current depression, M (SD)	47.7 (6.3)
Age at debut of consumption of alcohol, M (SD)	14.0 (4.8)
Age at first alcohol intoxication, M (SD)	15.7 (3.9)
Age at debut of regular alcohol consumption, M (SD)	40.9 (11.6)
Age at debut of perceived problematic alcohol consumption, M (SD)	40.9 (9.5)
DSM-5 diagnoses, *n (%)*	
AUD	7 (100%)
MDD	7 (100%)
Additional comorbid diagnoses, *n* (%)	5 (71%)
Country of birth, *n* (%)	
Sweden	6 (86%)
Other	1 (14%)
Highest level of education, *n* (%)	
University ≥ 3 years	1 (14%)
University ≤ 3 years	2 (29%)
Upper secondary school	1 (14%)
Other	3 (46%)
Source of income, *n* (%)	
Employed	3 (43%)
Unemployment benefits	1 (14%)
Sick leave	3 (43%)
Civil status, *n* (%)	
Married/cohabiting	3 (43%)
Single	4 (57%)

AUD, Alcohol Use Disorder (1); DSM-5, the Diagnostic and Statistical Manual of Mental Disorders, fifth Edition (American Psychiatric Association, 2013) (1), assessed with the MINI International Neuropsychiatric Interview, Swedish Translation Version 7.0.0 [MINI-7; (26)]; MDD, Major Depressive Disorder (1).

Prior to the treatment, a workshop was held by author OM, covering the treatment rationale and content. The treatment (see below), consisted of nine two-hour group sessions, held at the outpatient unit. Two clinical psychologists, authors AP and DWF, acted as therapists in the study. In addition to these treatment sessions, each participant had a 15-minute, individual weekly meeting with a therapist. These meetings were held to complete weekly measures, but also as a safety precaution, in case a patient relapsed or began to exhibit serious symptoms, for example increased suicidal thoughts. Authors AP, DWF, AB or AW held individual meetings. Follow ups were conducted in conjunction with the final session and three and six months later, by clinical psychologists not otherwise involved in the study. Participants received gift certificates of 50 SEK (approximately 5 USD) after each session and of 100 SEK (approximately 10 USD) post follow ups.

### 2.3 Treatment

The integrated treatment was originally developed for a homeless patient population with co-occurring MDD and SUD/AUD [see (17)]. In the current study, the treatment was adapted to an outpatient group format for patients with MDD and AUD. The integrated treatment model is built on a set of clinical assumptions. First, struggling with MDD and AUD implies having decreased activities within several important life areas, and alcohol use might be the one of the few pleasurable behaviors left for the individual. Second, both conditions include depressive symptoms, such as negative affect, and related avoidance-based strategies, including alcohol use, passivity, isolation, and avoidance of social contact. Third, when decreasing alcohol use, a transient ≈3 month period called post-acute abstinence or protracted abstinence may occur [see for example (27)]. Here, the individual experiences increased “depression-like” symptoms, and for those who previously used alcohol as a short-term strategy to cope with such symptoms, this period might be specifically related to alcohol lapses and relapses. Consequently, the overall aims of the integrated treatment are to (1) decrease alcohol use according to the patient’s own treatment goals (2); learn strategies to cope with negative affect; and (3) gradually increase meaningful activities such as work, social contact, exercise, or leisure activities, and learn strategies to cope with life changes. The integrated treatment includes psychological interventions from behavioral activation (28, 29) and relapse prevention (30), which are two evidence-based cognitive behavioral treatments for MDD and AUD, respectively. In the current study, additional interventions targeting rumination and alcohol cravings were included, which were not part of the original treatment protocol [see (17)]. In addition, psychoeducation on the disorders, their co-occurrence and interaction is included. Each session addresses both MDD and AUD. See **Table 2** for an overview of the treatment. For more information, contact the corresponding author.

**Table 2:** Overview of the integrated treatment.

Session	Content
1	Introduction, model of comorbidity for AUD and MDD. Rationale for CBT and integrated treatment. Treatment goals, including abstinence or controlled drinking. Homework: Treatment goals.
2	Psychoeducation: Lapse/relapse, post-acute abstinence, how MDD and AUD affect each other, risk situations/warning signals. Introduction to activity monitoring, controlled breathing – urge management. Homework: Practice controlled breathing. Activity monitoring. Read information about lapse/relapse.
3	Introduction to Martell’s model of depression and behavioral activation^a^. Activity scheduling, pleasurable stimulus control. Homework: Practice controlled breathing. Activity monitoring. Activity scheduling. Write down own risk situations. Read information about Martell’s model.
4	Psychoeducation: How AUD and MDD develop, triggers and self-medication. Introduction to functional analysis. Negative emotions, avoidance and lapse/relapse. Homework: Practice controlled breathing. Activity monitoring. Activity scheduling. Functional analyses.
5	Psychoeducation: Negative automatic thoughts, rumination and craving. Neurology, AUD and MDD. Homework: Practice controlled breathing. Activity monitoring. Activity scheduling. Functional analyses.
6	Psychoeducation and functional analysis: Strategies for managing rumination and craving. Homework: Practice controlled breathing. Activity monitoring. Activity scheduling. Functional analyses. Management of rumination/craving.
7	Functional analysis: Emotional regulation and lapse/relapse. Homework: Practice controlled breathing. Activity monitoring. Activity scheduling. Functional analyses. Maintenance plan.
8	Managing lapse/relapse. Early warning signs/risk situations. Maintenance plan. Homework: Practice controlled breathing. Activity monitoring. Activity scheduling. Review treatment goals.
9	Repetition of techniques learned during treatment. Evaluation of treatment and own goals. Set personal goals for the future.
Individual meetings in conjunction with each session	Monitor lapse/relapse. Reinforce sobriety/controlled drinking. Problem-solve potential homework difficulties. Complete self-report measures. Alert staff in case of relapse or other serious symptoms.

a = See Martell (29).

### 2.4 Statistical analyses and data preparation

**Figure 1:**
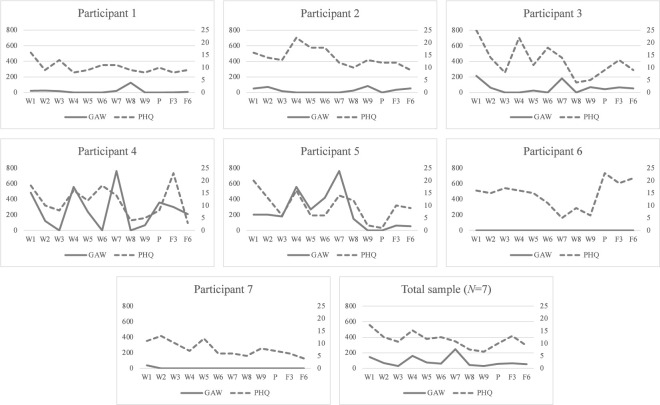
Individual alcohol consumption and depressive symptoms. Descriptive measures of GAW and PHQ-9 scores across study measure points. GAW, Grams of alcohol consumed per week; PHQ-9, Patient Health Questionnaire, total score (21); W, Treatment week, week 1 constitutes the baseline; P, Post treatment; F3, Three month follow up; F6, Six month follow up.

Statistical analyses were performed in R studio 1.4.1717 and Jamovi 2.3.28.0 (31, 32). Analyses were performed using intention-to-treat. As previously mentioned, one participant was classified as a drop-out as she only participated in the first treatment session and was therefore excluded from the analyses. Missing values in weekly measures (15%) were replaced using last observation carried forward (33) for descriptive purposes (see **Figure 1** and **Table 3**). Missing data in post and follow-up outcome measures (13%), were handled using total mean substitution (34). Changes in depressive symptoms (PHQ-9 total score), grams of alcohol consumed per week and HDD, respectively, were tested using the non-parametric Wilcoxon signed-rank test, to account for the small sample size. These analyses included baseline, post, three and six month follow up measures, and within-group effect sizes estimated using Rank biserial correlation (*r*) (35). PEth outcomes were presented at baseline and follow ups, as *n* participants above the clinical cut-off 0.30 μmol/l PEth, which indicates harmful drinking (36).

**Table 3:** Depressive symptoms and alcohol consumption across measurement points (N=7).

Measurement point	Depressive symptoms (PHQ-9 score), M (SD)	Grams of alcohol consumed per week
Session 1 (pre)	17.43 (4.31)	144.57 (172.63)
Session 2	12.57 (2.23)	68.57 (73.55)
Session 3	10.71 (3.82)	30.86 (66.31)
Session 4	15.29 (5.96)	160.00 (273.25)
Session 5	11.86 (3.89)	76.29 (122.71)
Session 6	12.57 (5.47)	60.00 (158.75)
Session 7	10.86 (3.85)	246.86 (359.08)
Session 8	7.57 (3.21)	42.86 (65.95)
Session 9	6.71 (3.45)	31.43 (39.56)
Post	10.00 (6.68)	57.71 (134.30)
3 month follow up	13.00 (6.06)	67.00 (106.72)
6 month follow up	9.14 (5.84)	54.00 (73.57)

## 3 Results

### 3.1 Treatment feasibility

The treatment was provided in routine outpatient AUD care. All current patients with MDD and moderate to severe AUD could be offered the treatment. The mean number of completed sessions was 7.71 (median=8, Sd=1.50). The proportion of completed sessions was 0.86, which was higher than that previously reported [0.70; see (17)]. The proportion of participants who completed all sessions was 0.43. It was lower than that previously reported [50%; see (17)]. One participant (12.5%) dropped out of treatment. This proportion was lower than that previously reported [20%; see (17)].

### 3.2 Credibility and satisfaction

Mean CEQ baseline ratings of credibility and expectancy, were 7.18 (Sd = 0.32, range 1-9) and 68% (Sd = 16.90, range 0-100%), respectively. Satisfaction with treatment at post was rated in the higher range of the CSQ-8, with a mean of 27.29 (Sd = 0.50) out of 32.

### 3.3 Preliminary effects

See **Figure 1** and **Table 3** for individual depressive symptoms and alcohol consumption during the study.

#### 3.3.1 Depressive symptoms

There were no significant reductions in the primary measure depressive symptoms from baseline to post, or baseline to 3 month follow up. From baseline to six month follow-up, depressive symptoms decreased significantly, with a small within-group effect size (r = 0.08) (see **Table 4**).

**Table 4:** Preliminary within-group effects (N=7).

Outcomes	Effects					
	Pre to post		Pre to 3 month follow up		Pre to 6 month follow up	
Primary	*P* value	Effect size (*r*)	*P* value	Effect size (*r*)	*P* value	Effect size (*r*)
Depressive symptoms	0.05	0.71	0.06	0.68	0.03*	0.08
Grams of alcohol consumed per week	0.02*	1.00	0.02*	1.00	0.03*	0.90
Secondary						
Heavy drinking days	0.02*	1.00	0.03*	0.90	0.02*	1.00

Wilcoxon signed-rank test Measure 1 > Measure 2; *r* = Rank biserial correlation; * = Significance level *p* <.05; Grams of alcohol consumed per week, derived from the Time Line Follow Back [TLFB; (22)]; Depressive symptoms, measured with the Patient Health Questionnaire [PHQ; (21)]; Heavy drinking days, percentage of days consuming >40 gram alcohol, derived from the Time Line Follow Back [TLFB; (22)].

#### 3.3.2 Alcohol consumption

There was a significant reduction in the primary measure grams of alcohol consumed per week, from baseline to post and follow-ups, with large within-group effect sizes (*r* range 0.75 to 0.90 to 1.00). The secondary alcohol measure, HDD decreased significantly between baseline, post and follow ups, showing moderate to large within-group effect sizes (*r* range 0.90 to 1.00) (see **Table 4**). The biomarker PEth was mainly consistent across measure points. At baseline 3/7 participants were above the clinical cut-off of 0.30 μmol/l PEth, and at post, three-, and six-month follow-ups, 3/7, 2/6, and 3/6, respectively.

### 3.4 Negative effects

No adverse events were reported during treatment at the weekly individual meetings. Self-reported negative effects were reported by six of seven participants post treatment, and the mean *n* of reported negative effects per participants was 4.29 (SD = 4.39) out of 32 using the NEQ. In total 30 negative effects were reported by the participants at post treatment, and 20 and 25 negative effects at three and six month follow up, respectively. In terms of content, the reported negative effects typically consisted of increased adverse emotional experiences, such as unpleasant memories, stress, worry or anxiety, but also included other experiences, for instance stopping believing that things would get better. See **Table 5**, for types of negative effects reported by more than one participant.

**Table 5:** Negative effects, reported by more than one participant.

Type of negative effect (NEQ)	Reported by *n* participants		
	Post treatment	Three month follow-up^a^	Six month follow-up^a^
Experienced unpleasant memories of previous life events	6/7	4/6	–
Felt more stress	3/7	2/6	2/3
Felt more sad	3/7	–	2/3
Stopped believing that things would get better	2/7	–	–
Felt afraid that others would understand that I received treatment	2/7	–	–
Experienced increased worry	2/7	–	2/3
Experienced increased anxiety	–	2/6	2/3
Felt more depressed	–	2/6	–

a = Follow up had missing data due to attrition, e.g., not all participants were assessed at each study timepoint. NEQ, Negative Effects Questionnaire (25).

## 4 Discussion

This study aimed to investigate if group treatment for co-occurring MDD and moderate to severe AUD in women, could be conducted at an outpatient unit within the Swedish public healthcare system. Overall, we conclude that it was feasible to deliver the integrated treatment in a naturalistic clinical context, and that the integrated treatment continues to show promise.

Treatment for co-occurring MDD and AUD is an emerging field (37). This study is one of the first of its kind as it investigates integrated group treatment based on evidence-based methods, was conducted in routine outpatient AUD care and for women specifically. The results are therefore difficult to compare to previous research. In terms of treatment feasibility, the proportions of completed sessions and drop outs were similar or better than previously reported (17). The study by Molander et al. (17) evaluated the integrated treatment in a face-to face format adapted to the specific needs of a homeless population, and therefore it might have been a poor comparison due to the differences in population. However, the current study showed comparable adherence measures to previous studies (16, 38), indicating good treatment feasibility. The treatment was rated high in credibility and satisfaction. This is important as offering acceptable and consumer friendly treatments has been shown vital to attract patients to treatments for AUD (39). Although the study design did not permit causal conclusions, preliminary effects were promising. Self-reported depressive symptoms and alcohol consumption were both reduced from baseline to follow ups. A few previous studies have evaluated integrated treatment approaches for depression and alcohol problems/misuse, and reported similar results (15, 16, 38, 40, 41). Overall, this indicates that depressive symptoms and alcohol consumption can be treated simultaneously, which might constitute an advantage. Carroll, Nich, and Rounsaville (42), found that when treatment for depression was added to substance use treatment, cocaine abusers with depressive disorders had better treatment retention and outcomes than cocaine abusers without such co-occurrence. In the current study, the difference between depressive symptoms at baseline and three month follow up was not significant. This is in line with research suggesting that happiness and self-esteem initially drop following the resolution of alcohol and drug problems (18). Compared to self-reported alcohol consumption, PEth measures remained unchanged from baseline to follow ups. A possible explanation may be lack of power due to the small sample size. Also, the PEth cut-off used in the study has been suggested as a clinical threshold for harmful drinking (36), but from a psychometric perspective it is less clear how PEth cut-offs relate to clinical change. Finally, negative effects were reported by most participants. Again, this may be due to potential decreases in happiness and self-esteem after the resolution of alcohol problems (18), that patients recovering from AUD, in our experience, often report regret, guilt and shame upon realizing how their alcohol use has affected them and their loved ones, or to post-acute abstinence (27). However, there is no established consensus on how to report and evaluate negative effects for psychological treatments [see (43)], but we note that most negative effects reported by participants, were increased adverse emotional experiences. Again, it could be argued that increased symptoms of depression, anxiety and stress, are a natural part of co-occurring MDD and AUD (especially when decreasing alcohol use), and that this is one of the main clinical features that the integrated treatment model was developed to address. In the current female sample the proportion of additional DSM-5 comorbid diagnoses was high, especially posttraumatic stress disorder (PTSD) (1). Therefore, it was not surprising that the most common reported negative effect was experiencing unpleasant memories of previous life events. This also highlights the need for additional integrated treatment approaches within routine AUD/SUD care, e.g., Concurrent treatment of Substance Use Disorders and PTSD using Prolonged Exposure (COPE) [see (44)].

The study has several strengths worth addressing. First and foremost, it was feasible to provide the treatment among treatment-seeking women with AUD and MDD in routine outpatient AUD care. No potential participants were excluded based on exclusion criteria. Participants shared characteristics with treatment-seeking men with MDD and SUD in another European country, e.g. that majorities were single, divorced or widowed and unemployed or on sick leave (45), suggesting that the participants in the current study may be representative of treatment-seeking individuals with MDD and SUD in Europe. Treatment was provided by clinical psychologists, commonly employed in outpatient AUD care, after very brief training. Taken together, this indicates that the investigated treatment, should it prove effective in RCTs, could be implemented in routine outpatient AUD care in a cost-effective manner given that existing staff can provide group treatment after very brief training. For these reasons, the study has high ecological validity, but perhaps more importantly, the study indicates that two prevalent and disabling psychiatric conditions may be treated concurrently when they co-occur. Second, both participants and treating psychologists were positive to the integrated treatment approach. Standard non-integrated treatment approaches have been criticized for trying to treat one diagnosis at a time (46). From a clinical perspective, assessment of comorbid MDD and AUD may be more complex than assessment of a single disorder. The treatment evaluated in the current study treats depressive symptoms and alcohol use simultaneously, and integrates key clinical processes from MDD and AUD, respectively. It was thus possible to treat patients with different symptom profiles across a clinical continuum of depressive symptoms and alcohol use (see **Figure 1**). Third, it was feasible to administer the integrated treatment in a group format. This treatment differed from other group treatments for AUD that had been delivered at the outpatient unit. Previously clinicians had judged it to be too risky to discuss participants’ individual alcohol use in a group setting, fearing that feelings of shame might trigger relapse or drop out, but in this study the decision was made to openly discuss depression and alcohol use in the group. Participants were very positive to this and expressed that it reduced feelings of shame and isolation. This is important as alcohol-related stigmatization is a barrier to treatment-seeking among individuals with AUD (47).

Some limitations should, however, be noted. This was a pilot study with few participants. Most participants in the study used antidepressant medication. To minimize potential confounders in the study medication doses were held constant during the integrated treatment, but interaction effects cannot be ruled out. Methods for handling of missing data were comparatively crude in the study. Multiple imputation (48) would have been preferable, but could not be used due to the low sample size/low variation in some variables. Regarding treatment limitations, the integrated model only assumes negative reinforcement (i.e., alcohol use to escape or relieve depressive symptoms), as a clinical maintenance process of MDD and AUD. Post-acute abstinence as a standalone factor for alcohol use has been criticized, as alcohol relapses and lapses also occur after the ending of this “depression-like” time period (27). Therefore, it may be worth considering whether additional features of clinical maintenance should be incorporated into the integrated treatment model, e.g., alcohol use as a positively reinforced behavior. Further treatment development could benefit from recent research on the clinical characteristics of patients with MDD and SUD, such as quality of life or sleep problems (49). Data were collected before the COVID-19 pandemic, but later research, carried out in Sweden, indicates that the pandemic had little effect on the need for AUD care and did not affect alcohol-related mortality (50, 51). European data indicates an increase in 100 percent alcohol-attributable mortality during the pandemic (52), something which may indicate an increased need for treatment for heavy drinkers in times of crises.

Future randomized controlled trials are needed to evaluate efficacy and effectiveness of the integrated treatment. It can be evaluated in relation to waiting list conditions, or to treatment as usual for patients with MDD and AUD in routine AUD/SUD care.

## 5 Conclusions

The newly developed integrated treatment for co-occurring depression and alcohol use disorder continues to show promise in terms of treatment feasibility, credibility, and satisfaction. Randomized clinical trials are needed to evaluate its effectiveness.

## Data Availability

The datasets presented in this article are not readily available because the authors do not have ethical consent from participants or the ethical review board to share data. Requests to access the datasets should be directed to OM, olof.molander@ki.se.
